# Charge-polarized interfacial superlattices in marginally twisted hexagonal boron nitride

**DOI:** 10.1038/s41467-020-20667-2

**Published:** 2021-01-12

**Authors:** C. R. Woods, P. Ares, H. Nevison-Andrews, M. J. Holwill, R. Fabregas, F. Guinea, A. K. Geim, K. S. Novoselov, N. R. Walet, L. Fumagalli

**Affiliations:** 1 Department of Physics & Astronomy, University of Manchester, Manchester, M13 9PL UK; 2 National Graphene Institute, University of Manchester, Manchester, M13 9PL UK; 3 Imdea Nanociencia, Faraday 9, 28049 Madrid, Spain; 4 Donostia International Physics Center, Paseo Manuel de Lardizabal, 4, 20018 Donostia-San Sebastian, Spain; 5 Centre for Advanced 2D Materials, National University of Singapore, Singapore, 117546 Singapore; 6 Chongqing 2D Materials Institute, Liangjiang New Area, 400714 Chongqing, China

**Keywords:** Ferroelectrics and multiferroics, Two-dimensional materials, Surfaces, interfaces and thin films

## Abstract

When two-dimensional crystals are brought into close proximity, their interaction results in reconstruction of electronic spectrum and crystal structure. Such reconstruction strongly depends on the twist angle between the crystals, which has received growing attention due to interesting electronic and optical properties that arise in graphene and transitional metal dichalcogenides. Here we study two insulating crystals of hexagonal boron nitride stacked at small twist angle. Using electrostatic force microscopy, we observe ferroelectric-like domains arranged in triangular superlattices with a large surface potential. The observation is attributed to interfacial elastic deformations that result in out-of-plane dipoles formed by pairs of boron and nitrogen atoms belonging to opposite interfacial surfaces. This creates a bilayer-thick ferroelectric with oppositely polarized (BN and NB) dipoles in neighbouring domains, in agreement with our modeling. These findings open up possibilities for designing van der Waals heterostructures and offer an alternative probe to study moiré-superlattice electrostatic potentials.

## Abstract (web summary)

Here, moiré superlattices generated by twisted insulating crystals of hexagonal boron nitride are shown to have a ferroelectric-like character, attributed to strain-induced polarized dipoles formed by pairs of interfacial boron and nitrogen atoms that create bilayer-thick ferroelectric domains.

## Introduction

One of the most promising avenues for controlling the properties of van der Waals (vdW) heterostructures is to adjust the angle between the stacked two-dimensional (2D) crystals. Such rotational control has allowed the observation of long-lived excitonic states^1^, resonant tunnelling^2,3^ and highly correlated electronic states^4–7^, including superconductivity in twisted bilayer graphene, among many other exciting effects, and various microscopic techniques have been shown to visualize moiré superlattices in twisted crystals^8–10^. At the same time, the twist-dependent electronic properties of hexagonal boron nitride (hBN), one of the most used crystals for engineering vdW heterostructures, have been overlooked so far. Like in the case of graphene, atomically thin crystals of hBN can be obtained through exfoliating the bulk material^11,12^. A wide-band insulator with strong polar covalent bonding between boron and nitrogen, hBN has proven itself indispensable for making high-quality vdW heterostructures^13,14^ and lateral superlattices, especially in combination with graphene^12,15–20^. Very recently, ferroelectric-like charge polarization has been observed on bilayer-graphene/hBN superlattices^21^. However, the possibility of generating a spontaneous charge polarization with the moiré superlattice of two twisted hBN crystals (twisted-hBN/hBN)—without graphene—has not been explored experimentally yet.

In this work, we demonstrate that hBN crystals placed at an intentionally small (‘marginal’) twist angle create a superlattice of charge-polarized macroscopic domains confined to the interface. The system undergoes reconstruction into a periodic commensurate phase that results in a high density of polarized interfacial dipoles between the hBN layers at the interface, as measured by electrostatic scanning probe microscopy^22^ at room temperature. We show that two dominant crystal alignments at 0° and 180° angle, referred to as parallel and antiparallel respectively, experience different reconstructions. Only the parallel alignment gives rise to ferroelectric-like domains, where aligned boron and nitrogen atoms at the interface layers create a dipolar field that reverses its sign in adjacent domains. Our conclusions are strongly supported by calculations of atomic reconstruction and charge density in the interfacial layer.

## Results

### Visualization of ferroelectric-like electrostatic domains

**Fig. 1:**
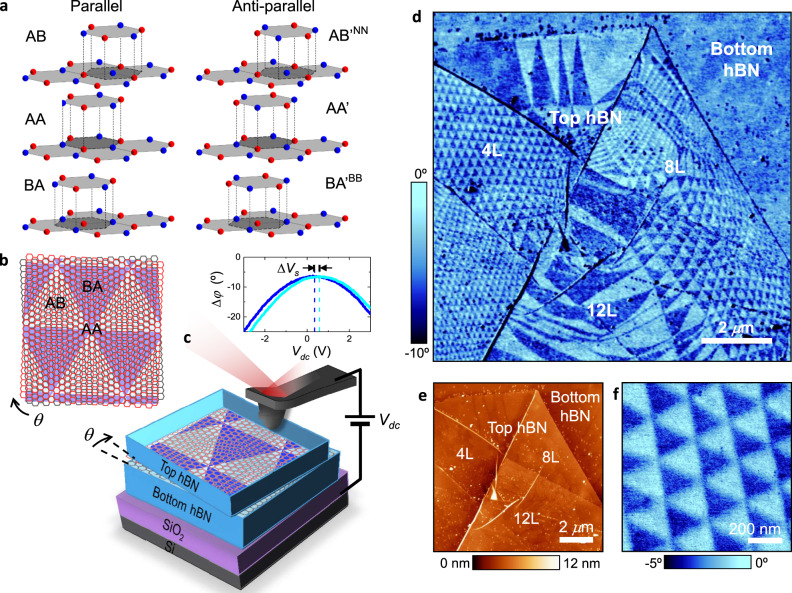
Electrostatic imaging of charge polarization in marginally twisted hBN. **a** Illustration of six high-symmetry stacking configurations for the hBN–hBN interface. Nitrogen atoms are shown in red; boron atoms in blue. **b** Schematic of adjacent hBN atomic layers (red and grey) misaligned by a small angle, *θ.* Dark and light triangles represent predominantly AB and BA regions, respectively. **c** Schematic of our experimental setup. Red and grey hexagonal lattices are in the top and bottom hBN, respectively. A voltage bias is applied between the AFM probe and the silicon substrate. Inset: representative dc-EFM curves as a function of the applied dc bias in two adjacent triangular domains. The horizontal shift of the maximum of the curves yields the variation in surface potential, ∆*V*_s_, between the domains. **d** Representative dc-EFM image (phase) of twisted hBN showing large areas with triangular potential modulation. Changes in domains’ shape and periodicity are due to small changes in *θ* caused by irregular strain and the wrinkles seen in the corresponding AFM topography image in **e**. The top hBN crystal has 4-, 8- and 12-layer thick regions. **f** Zoom-in of a region in **d** with regular domains.

Our heterostructures were made of a thin hBN crystal (1–20 layers) placed on top of a generally thicker hBN substrate (>30 layers) on highly doped Si/SiO_2_ substrates, following the procedures detailed in ‘Methods’ (also, see Supplementary Figs. 1 and 2). The two crystals were aligned at a twist angle *θ* < 1° and, for convenience, are referred to as top and bottom hBN, as depicted in Fig. 1b. We characterized the heterostructures using atomic force microscopy (AFM) and its electrostatic modes, electrostatic force microscopy (EFM)^23^ and Kelvin probe force microscopy (KPFM)^24^, which allow visualization of local electric charges by detecting variations in the surface potential (‘Methods’ and SI). EFM can be implemented with either dc or ac voltage bias between the tip and the Si substrate, which we refer to as dc-EFM^25^ and ac-EFM^26^, respectively (Supplementary Fig. 3). Below we focus on results obtained using dc-EFM for its high spatial resolution and simplicity of implementation. In this case, electrostatic domains can be detected directly in the AFM phase image by applying a dc bias, which allows faster scanning as compared to other electrostatic modes. ac-EFM and KPFM, which are generally slower and more demanding (they require to record an additional image using a second lock-in and, in the case of KPFM, a second feedback control^24^), yielded the same information (see Supplementary Figs. 4–9).

Figure 1 shows representative images taken from one of our twisted-hBN samples, in which the top hBN crystal has regions of 4-, 8- and 12-layer thickness (also see Supplementary Fig. 2). No superlattice pattern could be detected in the topography image (Fig. 1e) using standard contact and intermittent-contact modes. On the other hand, the corresponding dc-EFM image (Fig. 1d) reveals a clear pattern with triangular domains that are periodically arranged in many regions. The observed patterns do not extend beyond the area covered by the top hBN, indicating that they originate at the interface between the hBN crystals. This is also consistent with the fact that no pattern was detected on bubbles with contamination trapped between the two hBN crystals^27^ (Supplementary Figs. 4 and 5). Note that EFM-imaging is sensitive to subsurface properties^28,29^ (in our case, this is the interface between the hBN crystals) thanks to the long-range nature of electrostatic forces. The marginally twisted hBN shows large areas with triangular domains (Fig. 1f). However, as the size of domains increases, the moiré pattern becomes irregular. This can be understood by noticing that wrinkles seen in our AFM images are likely to induce local inhomogeneous strain and, for small twist angle (*θ* < 0.5°), even minor strain variations lead to large changes (∝ 1/*θ*) in the moiré periodicity^30^. Upon changing the sign of the applied dc bias, the contrast inverted (Supplementary Fig. S7), indicating that the observed pattern originates in permanent, built-in surface charges (as opposed to changes in dielectric properties^26,31^, see Supplementary Fig. 5). This was further confirmed by taking dc-EFM curves as a function of the bias (Fig. 1c, inset). The curves measured on adjacent domains are shifted with respect to each other by the potential difference, Δ*V*_s_ (see ‘Methods’ and SI), which was found to be the same Δ*V*_s_ ≈ 240 ± 30 mV in all our samples, irrespectively of the domain size (down to ∼30 nm), shape and orientation (see below). Additional KPFM images confirmed these results (see Supplementary Fig. 9).

The triangular patterns observed in our twisted-hBN structure resemble those observed in twisted bilayer graphene^32–34^. In the latter case, the triangular domains are alternating regions of AB and BA stacking. The case of hBN is more complex because its unit cell has two nonidentical sublattices, which leads to two cases of perfect alignment, parallel and antiparallel. Figure 1a illustrates the resulting six high-symmetry stacking configurations for an hBN–hBN interface. The parallel configuration allows AA, AB and BA domains. AB and BA are equivalent, except for the layer inversion such that the boron atoms align with the nitrogen atoms residing in either top or bottom layer, respectively (Fig. 1a). Because AB/BA alignment is more energetically favourable than AA^35,36^, it is expected that, if the local crystal strain is allowed to relax, triangular domains in Fig. 1b should have predominantly AB and BA stacking. The antiparallel configuration can form AAʹ, ABʹ^NN^ and BAʹ^BB^ domains, where ABʹ^NN^ and BAʹ^BB^ refer to the stacking where interfacial nitrogen is aligned with nitrogen and boron with boron, respectively (Fig. 1a). For small twist angles, regions of mixed stacking may occur but, as AAʹ stacking is more favourable than ABʹ^NN^ and BAʹ^BB^ (refs. ^35,36^), once the system relaxes, we expect hexagonal domains of predominantly AAʹ stacking (see below).

### Experimental determination of interfacial alignment

**Fig. 2:**
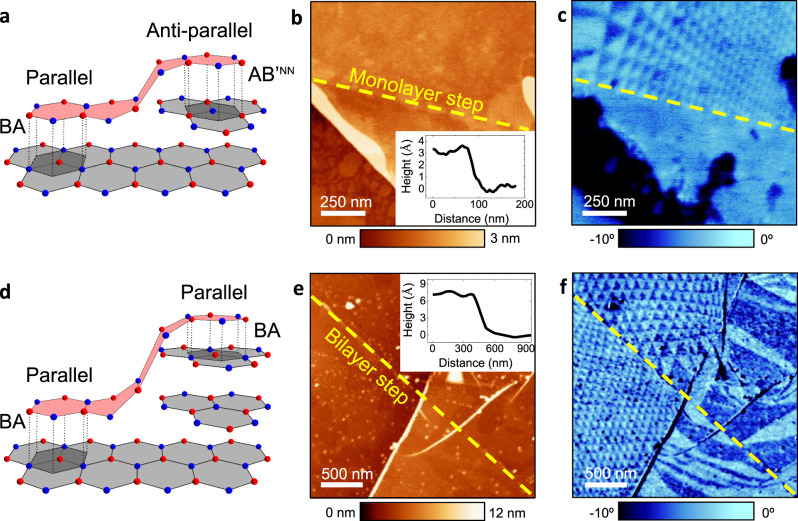
Effect of mono- and bi-layer terraces on occurrence of charge-polarized domains. **a** Illustration of hBN alignment over a monolayer terrace in the bottom hBN. The terrace forces an alignment change from parallel (left) to antiparallel (right) at the interface between top hBN (light red) and bottom hBN (light grey; AAʹ stacking). Dark-grey areas indicate BA and ABʹ^NN^ stacking. **b** AFM topography image of a representative sample, showing an hBN bilayer crystal covering a monolayer terrace in the bottom hBN. Inset: height profile across the step. **c** Corresponding dc-EFM image. The triangular potential modulation is visible only on one side of the step, marked by the yellow dashed lines in **b** and **c**. **d** Schematic as in **a** but for a bilayer terrace. The terrace in the bottom hBN (AAʹ stacking) does not influence the parallel alignment of the top hBN. The dark-grey shaded areas indicate BA stacking. **e** AFM topography image of an hBN crystal covering a bilayer step in the bottom crystal (inset: the step profile). **f** Corresponding dc-EFM image. The triangular modulation is visible on both sides of the step marked in yellow.

To determine whether the observed domains are a result of the parallel or antiparallel interfacial configurations, we fabricated and studied marginally twisted heterostructures with monolayer steps on the surface of the bottom hBN crystal. Crystallographically, this guarantees the presence of both parallel and antiparallel alignments within a single heterostructure device (Fig. 2a). Because bulk hBN naturally has AAʹ stacking, a monolayer terrace effectively produces rotation by 180° with respect to the adjacent region, as illustrated in Fig. 2a. Therefore, by aligning the top hBN over the single-layer terrace, one can probe both parallel and antiparallel configurations on the same sample, as illustrated in Fig. 2a—BA stacking on the left side and ABʹ^NN^ stacking on the right side. Figure 2b, c shows AFM and EFM images for a sample with top-hBN aligned over a monolayer step indicated with the dashed yellow line. The topography image in Fig. 2b shows that the terrace is formed by a monolayer (*h* ≈ 0.33 nm). In the corresponding dc-EFM image (Fig. 2c), we observe a clear triangular pattern on one side of the terrace which cuts off sharply at the terrace edge, with no periodic signal on the other side. This suggests that periodic domains are possible only for one orientation type, either parallel or antiparallel. We repeated this experiment on several samples, confirming the generality of the observation (Supplementary Figs. 4 and 6). To corroborate this further and rule out the possibility that monolayer terraces facilitate a rotation or some other changes that prevent the observation of the charge-polarized domains on one of the sides of monolayer steps, we also fabricated samples with bilayer terraces (*h* ≈ 0.66 nm). This crystallographic arrangement is shown schematically in Fig. 2d. It is clear that bilayer steps should make alignment identical in the neighbouring regions (Fig. 2d shows the case of BA stacking). In agreement with the expectations, we found the ferroelectric domains on both sides of bilayer steps with no difference in the contrast (Fig. 2e, f).

**Fig. 3:**
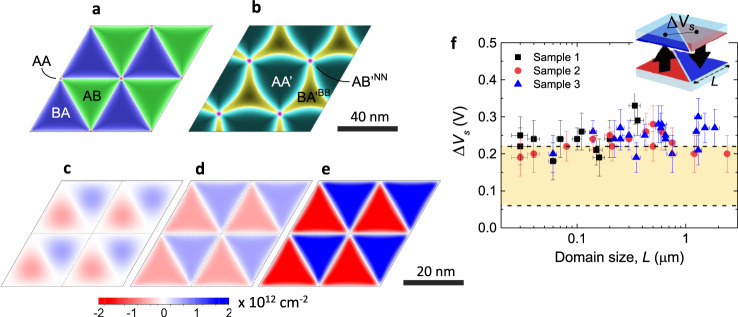
Calculated charge-density distribution in marginally twisted hBN. **a**, **b** Dominant stacking order for twisted-bilayer hBN, calculated as in refs. ^47,52^, for *θ* = 0.33° in the case of parallel (**a**) and antiparallel (**b**) alignment. AB staking is shown in dark green, BA—dark blue, AA—red, AAʹ—dark cyan, BAʹ^BB^—dark yellow and ABʹ^NN^—magenta. The AA and ABʹ^NN^ alignments occur at the intersections of the AB and BA regions, and AAʹ and BAʹ^BB^ regions, respectively. The colour intensity indicates the degree of alignment of boron and nitrogen atoms located in the two hBN monolayers. The atoms are perfectly aligned in the domains’ centres. Scale bar: 40 nm. **c**, **d**, **e** Charge-density distribution within individual hBN monolayers, which is induced by interlayer interaction for the case of parallel alignment for *θ* = 0.52°. Scale bar: 20 nm. **c** Relatively weak interlayer hopping without lattice relaxation. **d** Same hopping but accounting for the lattice relaxation. **e** Stronger hopping with lattice relaxation. Twisted bilayer hBN remains charge-neutral, and the charge polarity is reversed between the two layers (red and blue reverse), which also reflects the inversion symmetry of AB/BA stacking. **f** Electrostatic potential variation in the centre of AB and BA, as illustrated in the inset. The experimental values (symbols) as a function of domain size, measured at scan height *h*_s_ = 8–12 nm from the domains plane. The *x*-axis and *y*-axis error bars show the uncertainty of the technique in lateral size and surface potential. Within our accuracy, the potential is size-independent. The yellow-shaded region denotes the calculated surface potential values delimited by the two hopping amplitudes used in **d**, **e**.

It is important to note that the observed potential contrast is independent of the superlattice periodicity, as shown in Fig. 3f. The contrast remained constant down to the smallest domains, of ~30 nm in size, that we could detect within our lateral resolution (‘Methods’ and Supplementary Fig. 8). This observation rules out the possibility that the potential pattern can arise from piezoelectricity effects as a result of in-plane deformation, as reported for hBN previously^12^. Indeed, the piezoelectric charge should be proportional to the strain gradient^37^ and, accordingly, strain variations are expected to decrease with increasing the domain size, in contrast to the experimental results. Our analysis also shows that strain gradients and thus the piezoelectric charge should be localized at the domain edges (Supplementary Fig. 10). Furthermore, the observed contrast was found to be practically independent of the thickness of top and bottom hBN crystals for the investigated range of thicknesses. We detected the contrast in all our samples with top hBN ranging from a monolayer up to 18 layers (6 nm) whereas bottom hBN was up to 80 nm thick. This independence is of practical importance because it greatly simplifies access to the charge-polarized twisted superlattices, without the need of using mono- or few-layer crystals, which is experimentally challenging for the case of hBN^11^. We note, however, that for thick hBN crystals, atomic relaxation at the interface is generally expected to be hindered by an additional elastic contribution from the bulk, which may result in weaker interfacial strain and somewhat smaller polarization (see below).

### Theoretical lattice reconstruction and charge distribution

To quantify our experimental observations, we calculated superlattice reconstructions at the twisted hBN interface, focusing on the case of marginally twisted monolayers. Atomic reconstructions occur as a result of the balance between the energetically favourable interlayer alignment and the elastic energy required to reach the relaxed lattice configuration. Figure 3a, b shows the stacking order after relaxation for a twist angle of 0.33° for parallel and antiparallel alignments, respectively. In all cases, the observed superlattices form periodic commensurate states with superlattice periodicity matching that of the moiré pattern between them. The antiparallel alignment forces the superlattice to relax into a rather complex configuration with threefold symmetry (Fig. 3b), maximizing the area of AAʹ stacking between the two crystals whilst minimizing the unfavourable ABʹ^NN^ and BAʹ^BB^ stacking. In contrast, the parallel interfacial alignment relaxes into a much simpler triangular superlattice (Fig. 3a), where the AA aligned region becomes very small. The latter maximizes the total area of the inversion-symmetric AB/BA stacking that is energetically favourable with respect to AA stacking^36^. Comparison between the calculated shapes and our EFM images strongly suggest that the observed superlattices represent the case of parallel alignment with alternating AB and BA domains. Next, we calculated a charge-density distribution within the domains. This required full details of all energy bands in our tight-binding model (details in SI). The calculations show that even without lattice reconstruction, a considerable charge polarization occurs at the interface (Fig. 3c). However, to obtain triangular domains with sharp boundaries such as those observed in the experiment, the lattice relaxation is needed to be accounted for, as it enhances crystallographic alignment over the entire unit cell (Fig. 3d). A large charge-density modulation was found only for the parallel alignment (Fig. 3d, e). For the antiparallel one, our calculations yielded negligible surface charge densities, three orders of magnitude smaller than in the case of parallel alignment. This small polarization would be dwarfed by piezoelectric effects induced by in-plane elastic strain. The strong potential modulation observed experimentally is another proof of the parallel rather than antiparallel alignment in marginally twisted hBN.

The interaction-induced charge density within hBN monolayers is not expected to vary with the domain size, in agreement with the experiment (as long as it is larger than the reconstructed region at the domain wall—approximately 4 nm according to our calculations). On the other hand, the absolute value of the surface charge was found to be very sensitive to interlayer hopping parameters. For the range of values reported in the literature, we obtained the charge densities around few 10^12^ cm^−2^ (see Fig. 3d, e). For the larger hopping parameters used in Fig. 3e, the density modulation reaches approximately ±3 mCm^−2^. Using the simple parallel-plate approximation and assuming interlayer dielectric constant of 1, the above value translates into a surface potential modulation of about 0.2 eV, in fair agreement with the experiment. Nonetheless, the comparison between the experiment and theory in Fig. 3f indicates that the theory probably underestimates the strength of interlayer interactions. Further work using advanced computational methods is required to clarify the interaction strength and the induced spontaneous charge polarization at the interface.

## Discussion

To qualitatively understand the physics behind our observations, let us consider aligned pairs of boron and nitrogen atoms located in the different hBN monolayers of the interface. Their interaction generates BN and NB dipoles aligned along hBN *c*-axis (out of plane). For AAʹ stacking (Fig. 1a), the dipoles have both polarizations (BN and NB) within the same unit cell. In contrast, for AB/BA stacking, only one orientation of dipoles (either BN or NB) is present, which breaks the symmetry along the *c*-axis and effectively creates a ferroelectric bilayer with a fixed polarization. The reversal between AB and BA stacking in adjacent domains leads to their opposite charge polarization, which is responsible for the surface potential difference imaged by EFM and KPFM.

In conclusion, we observed triangular dipolar domains in marginally twisted hBN that originate at the interface between the two hBN crystals, as in a bilayer-thick ferroelectric in the out-of-plane direction. The demonstrated charge-polarized superlattices provide a fascinating platform to study superlattice phenomena and engineer vdW heterostructures. The fact that a small twist angle between two insulating 2D crystals can generate a strong interfacial charge polarization of known amplitude and periodicity is an important addition to the arsenal of vdW technologies. The major opportunity is the formation of sharp (down to few unit cells sharpness due to lattice reconstruction) lateral potential steps. It can be used, for example, to create an artificial surface potential and spatially modify properties of adjacent 2D materials such as graphene or transition metal dichalcogenides (TMDs), and to engineer novel electronic and optical devices. For example, using twisted hBN as a dielectric layer in back-gated devices, we will produce atomically sharp p–n junctions which can be used to form light-emitting diodes in TMDs or complex devices to study the properties of 2D electron gas in 2D materials, such as focusing, Veselago lens^38^, etc. Another opportunity is the emergence of an interfacial dipole moment responsive to the external electric field, which may allow the use of such structures in piezoelectric actuators or ferroelectric memories. Our work is also important because it shows a simple and non-destructive scanning probe technique able to visualize moiré-superlattice electrostatic potentials, which is expected to be useful to study other vdW heterostructures, such as twisted TMDs bilayers.

We note that results supporting these findings were reported in two other works^39,40^ during the review of our manuscript.

## Methods

### Sample preparation

We fabricated twisted-hBN heterostructures on oxidized Si wafers (290 nm of SiO_2_) by using the PDMS/PMMA (polydimethylsiloxane/poly(methyl methacrylate) dry transfer technique^41,42^, described briefly here and further in SI. In short, we exfoliated hBN crystals onto the Si substrate and identified target crystals using optical microscopy (Supplementary Fig. 2). We chose the crystals on the basis of two requirements: first, two hBN crystals should be adjacent to each other and, second, the perspective bottom hBN should have a monolayer or bilayer terrace in its top surface. The first condition assured near-perfect alignment between the two crystals, creating zigzag-to-zigzag and armchair-to-armchair edges. Indeed, if the crystals are adjacent to each other, they are likely to split from the same original crystal. Without following this rule, there would be a 50% chance of creating 30° misaligned samples (zigzag-to-armchair alignment). The second requirement was used in the experiments discussed in Fig. 2, where we studied parallel and antiparallel alignment in a single device.

### AFM and electrostatic imaging

We acquired simultaneous AFM topography and electrostatic images using the standard two-pass method^22^. First, we measured topography in the dynamic mode by oscillating the tip at its free mechanical resonance with no applied electric field. In the second pass, the electrostatic signal was recorded while retracing the topography line with an applied voltage after retracting the tip a few nanometres. We measured the electrostatic signal with either dc or ac applied voltage. In dc-EFM^43^ (Supplementary Fig. 3a), also called phase-EFM, we applied a dc bias of 2–3 V and recorded the AFM phase shift, Δ*φ*, of the cantilever mechanical oscillations, which depends quadratically on the surface potential, *V*_s_. In ac-EFM^26^ (Supplementary Fig. 3b), we excited the cantilever with an ac voltage of amplitude 4–5 V and frequency *ω* = 1–10 kHz, and we recorded the amplitude variation of Δ*φ* at *ω* and 2*ω* using an additional lock-in amplifier. No triangular domains were detected at 2*ω,* indicating that the observed domains do not have a dielectric origin^26,31^ (Supplementary Fig. 5). We quantified the surface potential variation, Δ*V*_s_, in Fig. 3f by taking Δ*φ* versus dc voltage curves in the centre of the domains, as detailed in SI. KPFM images (Supplementary Fig. 9) were recorded by nullifying the amplitude of Δ*φ* at *ω* using a second feedback control and applying both dc and ac voltages, where the dc voltage is used to compensate a variation in *V*_s_ and thus obtain Δ*V*_s_ images. All the curves and KPFM images used to quantify Δ*V*_s_ were taken with the tip scan height, *h*_s_, in the range 8–12 nm from the dipolar domain plane, corresponding to the minimum stable value of *h*_s_ that we could use on all our samples. This is important because on the smallest domains, the built-in potential decays rapidly for larger values of *h*_s_ (Supplementary Fig. 8). We note that the force-gradient measurement approach employed here based on phase-shift detection is advantageous to minimize the impact of long-range forces as compared to conventional amplitude-modulation EFM or KPFM that measure the force, thus increasing the lateral resolution. To further increase it, we employed doped silicon probes (Nanosensors PPP-CONTR, spring constant 0.5–1.5 Nm^−1^) that have smaller tip radii (<7 nm) than conventional metal-coated probes. We thus estimate the lateral resolution in our experiments to be in the range 5–10 nm^44^. This agrees with our observations in which we quantified the built-in potential to be constant for triangular domains as small as 30 nm. We note that within our accuracy, the EFM contrast is independent of the domain size or shape, because in the experimental conditions used here the contrast originates from the short-range interaction of the tip with the surface area below and around the apex, which was much smaller than the lateral size of the domains analysed here. Care should be taken if electrostatic images are taken with larger tip radii or larger scan heights or on thicker top-hBN, as the observed built-in potential may be reduced and become size-dependent in the case of small domains (details in SI and Supplementary Fig. 8). Data were acquired and processed using WSxM software^45^.

### Theoretical calculations

The theoretical calculations were performed in two stages: first, we used LAMMPS^46^ to minimize the energy using a classical potential model for relaxation^47^, using the ‘inter-layer potential’ (ILP) from refs. ^48,49^ with the Tersoff in-layer potential^50,51^. The results were plotted using an extension of the method in ref. ^47^ where we took into account all six alignment options shown in Fig. 1. Using the found deformed lattice, we then performed tight-binding calculations using electronic coupling with exponential Koster-Slater interlayer hoppings. We used the standard in-layer nearest neighbour hopping of 2.33 eV (refs. ^36^), neglecting its modification by minor bond stretching. The charge density was then calculated by summing over all occupied states, which limited the smallest angle we could perform calculations for to about 1°. Details of these calculations as well as further results on the electronic structure of twisted hBN can be found in ref. ^52^.

## Supplementary information

Supplementary Information

## Data Availability

The data generated during this study are available from the corresponding authors on reasonable request.
