# Supplementary material for: Charge-polarized interfacial superlattices in marginally twisted hexagonal boron nitride
Source: Nat Commun. 2021 Jan 12;12:347. doi: 10.1038/s41467-020-20667-2 (PMC7804449; doi:10.1038/s41467-020-20667-2)
Supplement: Supplementary file 1 — Supplementary Information [file 41467_2020_20667_MOESM1_ESM.pdf]

Supplementary Information

Charge-Polarized Interfacial Superlattices in Marginally Twisted Hexagonal Boron Nitride

C. R .Woods[1,2]*, P. Ares[1,2], H. Nevison-Andrews[1,2], M. J. Holwill[1,2] , R. Fabregas[1], F. Guinea[3,4], A. K. Geim[1,2], K. S. Novoselov[1,2,5,6], N. R. Walet[1], L. Fumagalli[1,2]*

*Corresponding Authors: CRW – colin.woods74@gmail.com, LF - laura.fumagalli@manchester.ac.uk

[1]Department of Physics & Astronomy University of Manchester, Manchester, M13 9PL, UK

[2]National Graphene Institute, University of Manchester, Manchester, M13 9PL, UK

[3]Imdea Nanociencia, Faraday 9, Madrid 28049, Spain

[4]Donostia International Physics Center, Paseo Manuel de Lardizabal, 4, 20018 Donostia-San Sebastian, Spain

[5]Centre for Advanced 2D Materials, National University of Singapore, Singapore 117546, Singapore

[6]Chongqing 2D Materials Institute, Liangjiang New Area, Chongqing 400714, China

Supplementary Note 1. Sample fabrication

Twisted-hBN heterostructures were fabricated as schematically shown in Supplementary Fig. S1. First, the hBN crystals were isolated by mechanically exfoliating commercially available bulk crystals. The mechanical exfoliation was done using scotch tape to reduce the bulk material until the crystals were significantly thinner and more numerous. The resultant material was then deposited onto a SiO_2/Si (290 nm) substrate. SiO_2/Si substrates provide excellent optical contrast for even the thinnest flakes and terraces on their surfaces, particularly when combined with wavelength filtering, dark-field imaging and Nomarski filtering (see Supplementary Fig. 2). These techniques were used to identify pairs of adjacent hBN crystals: one thin crystal of 1-20 layers (top hBN) and one thicker crystal of more than 30 layers (bottom hBN). Importantly, the bottom hBN crystal was only used if it contained a monolayer or bilayer terrace in its surface (Supplementary Fig. 2b), as confirmed by AFM topography imaging (Supplementary Fig. 2c and Figs. 2b and 2e in the main text). Further, using adjacent hBN crystals removes the requirement to use crystallographic fractures as an alignment tool during the transfer process (a method limited to 50% probability of success). Because it is likely that the two crystals are from the same growth domain, they are already in near-perfect alignment. Once the pair of hBN crystals were found on the SiO_2/Si substrate , we brought them together using the PDMS/PMMA (Polydimethylsiloxane/Poly(methyl methacrylate)) dry-peel transfer technique[1]. The bottom hBN (light grey) and top hBN (light red) are identified on the SiO_2/Si substrate and a PDMS/PMMA membrane (green) is positioned above the relevant area using a micromanipulation stage (Supplementary Fig. 1a). The membrane is brought into contact with top hBN only (Supplementary Fig. 1b). Then the membrane is removed, lifting the top hBN crystal with it (Supplementary Fig. 1c). The membrane was then translated with no rotation, so that the top hBN crystal was above the terrace in the bottom hBN, and the two were brought into contact (Supplementary Fig. 1d). Finally, the membrane was removed, leaving the top hBN on the bottom hBN (Supplementary Fig. 1e). A photograph of our micromanipulation stage (from Graphene Industries) is shown in Supplementary Fig. 1f (see details in Ref.[2]).

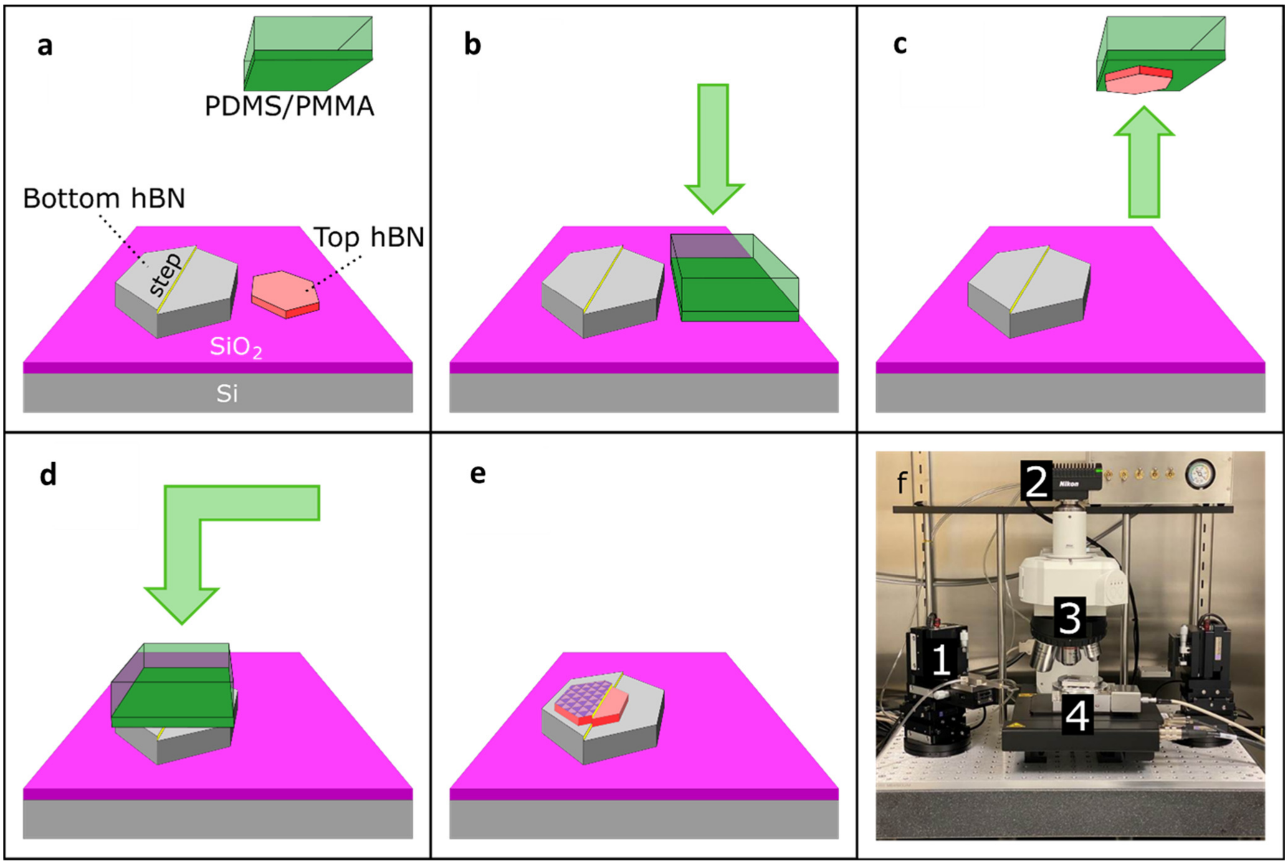

Supplementary Figure 1 | Schematic of the procedure used to fabricate marginally twisted hBN heterostructures. **(a)** Bottom (light grey) and top (light red) hBN crystals are identified on a SiO_2/Si substrate. The bottom hBN is confirmed to have a monolayer or bilayer terrace in its surface (yellow step). **(b)** A PDMS (faint green) and PMMA (green) membrane is brought into contact with the top hBN. **(c)** The membrane and top hBN are lifted from the substrate. **(d)** The membrane is translated above the bottom hBN with no rotation and brought into contact. **(e)** The membrane is removed leaving both crystals in place. **(f)** Photograph of our micromanipulation stage (from Graphene Industries). The labelled features are: (1) micromanipulation transfer arm for membrane movement, (2) camera for alignment, (3) optical magnification, and (4) stage micromanipulation and rotation – see details in Ref.[2]

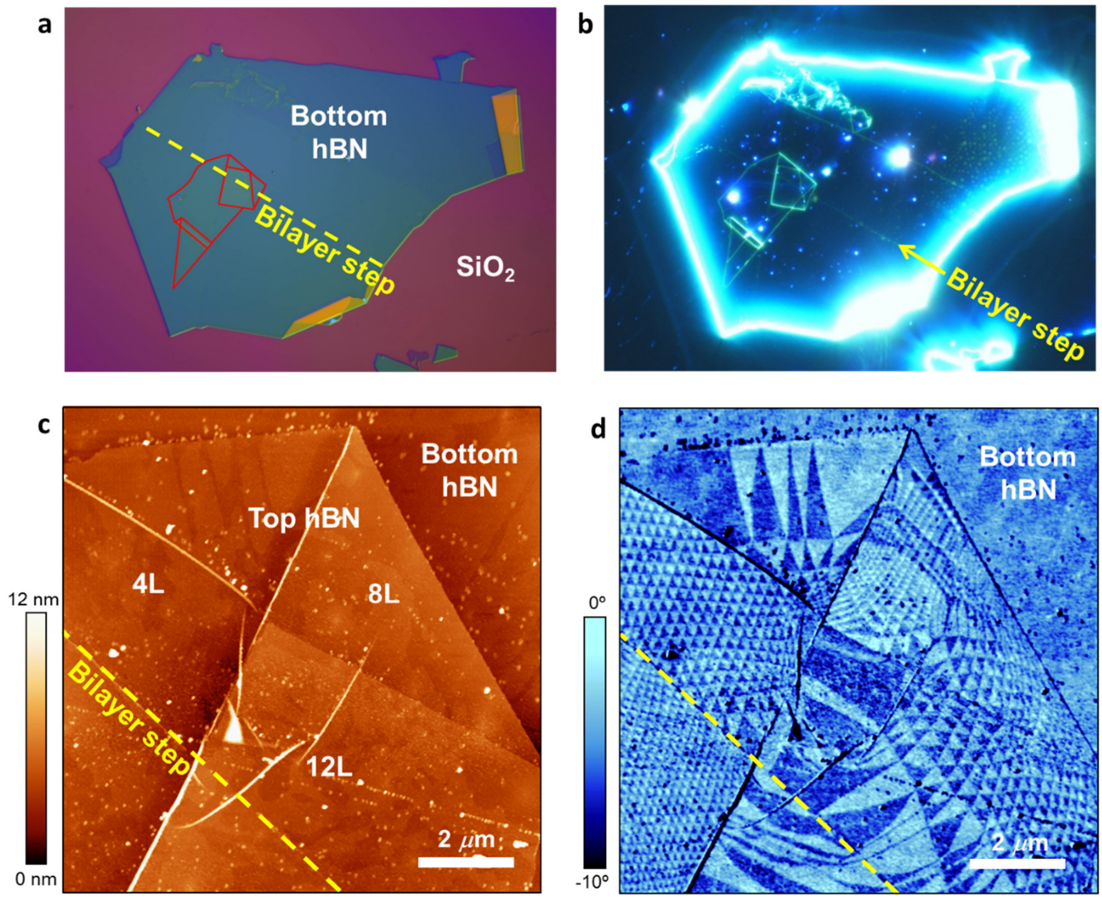

Supplementary Figure 2 | Optical and AFM images of marginally twisted hBN on terraces. (a,b) Representative optical images of one of our twisted samples, shown in Fig. 1d-f and Fig. 2e,f in the main text. The top hBN crystal (red line) was transferred on the bottom hBN crystal in a region with a bilayer step, marked by the yellow dashed line, which is visible in the dark-field image in **(b)**. **(c,d)** Corresponding AFM topography and EFM image of the top hBN in the region near the bilayer step (yellow dashed line). The top hBN crystal has 4-layer, 8-layer and 12-layer-thick regions. The triangular potential modulation is detected in all the regions, regardless of their thickness, and on both sides of the bilayer step.

Supplementary Note 2. Electrostatic imaging

The triangular potential modulation was detected using electrostatic force microscopy (EFM) and Kelvin probe force microscopy (KPFM). Both techniques are non-contact scanning probe techniques that probe the electrostatic interaction between a conductive AFM tip and the sample[3,4]. EFM measures local electrostatic force variations that can originate from either a variation in the surface potential or in the dielectric properties of the sample. In this section, we present additional information and images using EFM. KPFM is just an advanced EFM mode in which surface potential variations are recorded using an additional feedback loop, and we discuss it in the next section (see S3).

EFM images in the main text were taken using dc-EFM mode (also known as phase-EFM)[5] by simply applying a dc voltage, as illustrated in Supplementary Fig. 3a. The cantilever was oscillated at its free mechanical resonance with a dc voltage applied between the tip and the silicon substrate, and the phase shift, $\Delta\varphi$, of the mechanical oscillation of the cantilever was recorded while scanning the surface. The electrostatic force experienced by the cantilever can be written as $F_{el}(z) = \partial C/\partial z\,(V_{dc} - V_s)^2/2$, where C is the total tip-sample capacitance, V_{dc} is the applied dc voltage between the AFM tip and the sample substrate, V_s is the surface potential and z is the tip-surface distance. To a first approximation, the phase shift directly depends on the force gradient as $\Delta\varphi(z) = -\frac{Q}{k}\frac{\partial F}{\partial Z}$. Thus, in the presence of an electrostatic force, it can be written as $\Delta\varphi(z) = -\frac{Q}{2k}\frac{\partial^2 C}{\partial z^2}(V_{dc} - V_s)^2$ where Q is the quality factor of the cantilever and k its spring constant. Hence, the phase shift varies proportionally to the square of the tip-surface potential difference and to the second derivative of the tip-surface capacitance, $\partial^2 C/\partial z^2$. The latter is a complex function of the geometric and dielectric properties of the tip-sample system.

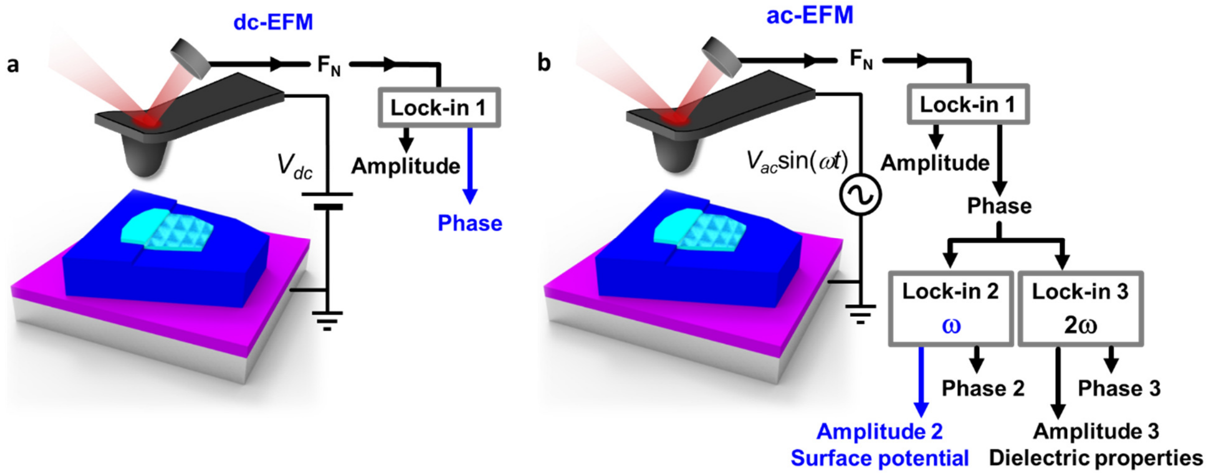

Supplementary Figure 3 | Schematics of the EFM setup. **(a)** In dc-EFM, the AFM phase was recorded during the second pass with a dc voltage applied between the AFM probe and the silicon substrate. **(b)** In ac-EFM, an ac voltage of frequency ω was applied between the AFM probe and the silicon substrate during the second pass. The amplitude of $\Delta\varphi(\omega)$, which depends on the surface potential, showed periodic triangular modulation on the twisted-hBN crystals, while the amplitude of $\Delta\varphi(2\omega)$, which depends only on the surface dielectric properties, showed no periodic pattern.

We recorded $\Delta\varphi$ using the standard two-pass method. First, we acquired the topography image with no applied voltage. Then we retraced it with an applied dc bias of 2-3 V, the AFM feedback control switched off and the tip lifted up a few nm with respect to the first pass. The lift height, z_{lift}, set in the range 3-5 nm, was chosen large enough to avoid short-range forces, but as small as possible to minimize the tip-surface distance, z. This is important to maximize the lateral resolution of the technique, which is set by the $\partial^2 C/\partial z^2$ term and decreases with the tip moving away from the surface. By keeping the oscillation amplitude in the range of 5-10 nm, we typically took the EFM images at a total scan height of 8-20 nm from the interface between the twisted hBN crystals. We note that by probing the force gradient through $\Delta\varphi$ instead of the force as in standard amplitude-modulation EFM or KPFM, the dc-EFM mode employed here is less sensitive to long-range forces from the tip cone and cantilever. Thus, it allows higher lateral resolution and it is advantageous here to study small domains. To discriminate whether the observed triangular pattern was a built-in potential or a change in the dielectric properties of the heterostructure, we took $\Delta\varphi$ curves as a function of the applied dc bias in the centre of the triangular domains, as detailed in section S3. They show the expected parabolic behaviour (see Supplementary Fig. 7)[6-8]. While the curvature, set by the capacitive term $\partial^2 C/\partial z^2$, was independent of the domain, the maximum of the parabola shifted a few hundreds of mV with the domain polarity. This allowed us to conclude that the triangular modulation detected in the EFM images is a built-in potential due to the interfacial charge distribution between twisted hBN crystals.

To support this conclusion, we also took images in ac-EFM mode, illustrated in Supplementary Fig. 3b, which allowed us to record dielectric images. In this mode, an ac voltage bias of frequency ω and amplitude V_{ac} was applied between the AFM tip and the sample substrate. This modulates the electrostatic force at ω and 2ω with amplitude $F_{el}(\omega) = \partial C/\partial z\, V_s \cdot V_{ac}$ and $F_{el}(2\omega) = \partial C/\partial z \cdot V_{ac}^2/4$, respectively. While the ω harmonic is again proportional to both the surface potential V_s and the dielectric properties through the capacitive term $\partial C/\partial z$, the 2ω harmonic depends only on the $\partial C/\partial z$ term[9]. Using two additional lock-in amplifiers, we measured the amplitude of both harmonics. Note that, also in this mode, we measured the phase shift $\Delta\varphi$ of the mechanical oscillation of the cantilever instead of the force, thus recording the two phase harmonics, $\Delta\varphi(\omega)$ and $\Delta\varphi(2\omega)$. Again, this is advantageous because $\Delta\varphi$ is proportional to $\partial^2 C/\partial z^2$ and therefore it allows higher spatial resolution. Supplementary Figure 4b shows a representative ac-EFM image at ω on one of our twisted hBN samples. The potential modulation extends over large regions with regular and irregular triangular domains, similarly as the one observed in dc-EFM in Fig. 1d-f, and only on one side of a monolayer step. Supplementary Figure 5 shows zoom-in ω and 2ω images in flat regions around bubbles filled with contamination. The domains are visible in the ω image, but not in the 2ω image which depends only on the surface dielectric properties. This confirms that the triangular domains reflect a built-in potential that originates at the interface between twisted hBN crystals, not a change in dielectric properties. Figure S6 shows additional images taken in regions around other monolayer steps. Again, they show small triangular domains only on one side of monolayer steps.

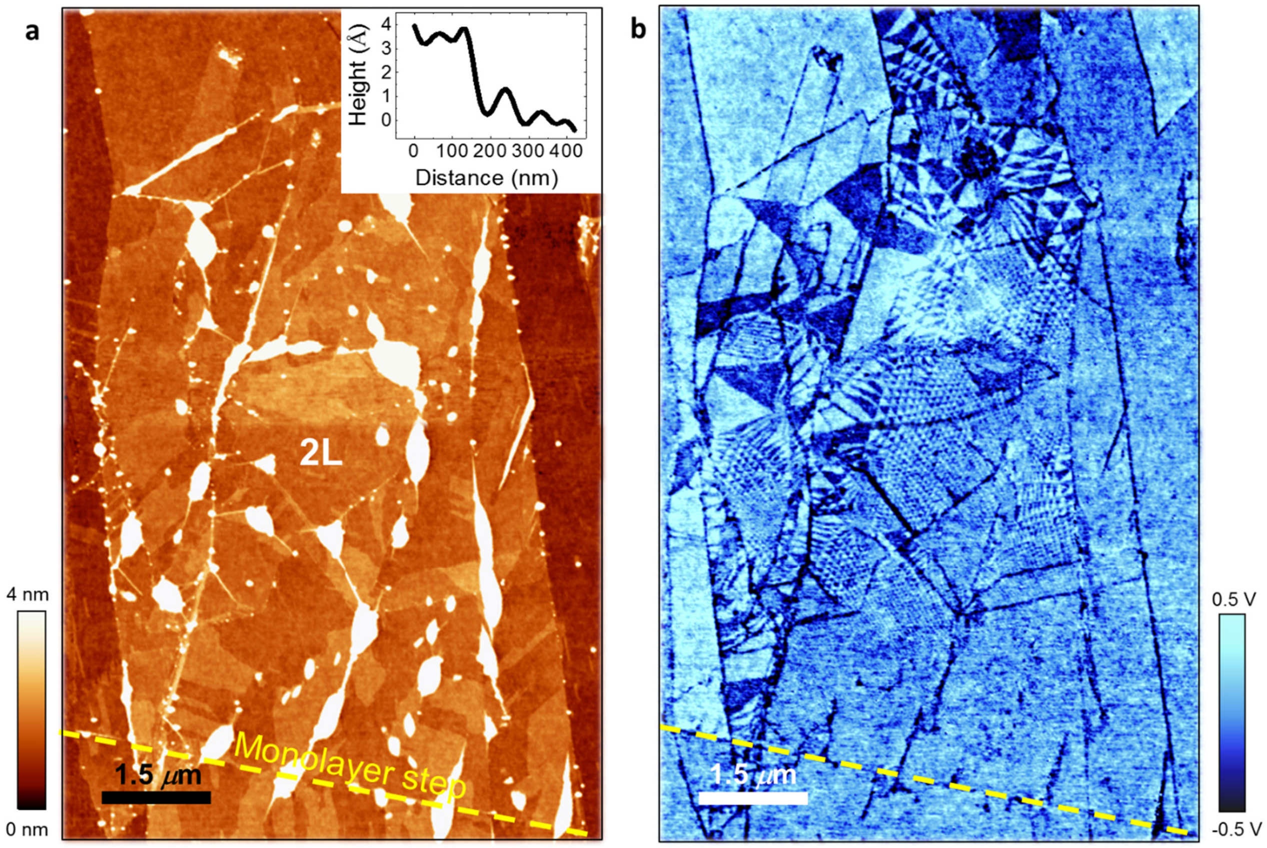

Supplementary Figure 4 | ac-EFM image of triangular potential modulation in marginally twisted hBN. (a) Representative AFM topography of a twisted-hBN sample and **(b)** corresponding ac-EFM image at ω. The colour scale in **(a)** was adjusted to show the details of flat regions instead of bubbles (white regions) that are filled with contamination and several nm high. Large areas with triangular potential modulation are visible in **(b)**, only on the flat regions and only on one side of a monolayer step marked by the yellow dashed lines. The inset in **(a)** shows a profile perpendicular to the monolayer step depicted by the yellow dashed line.

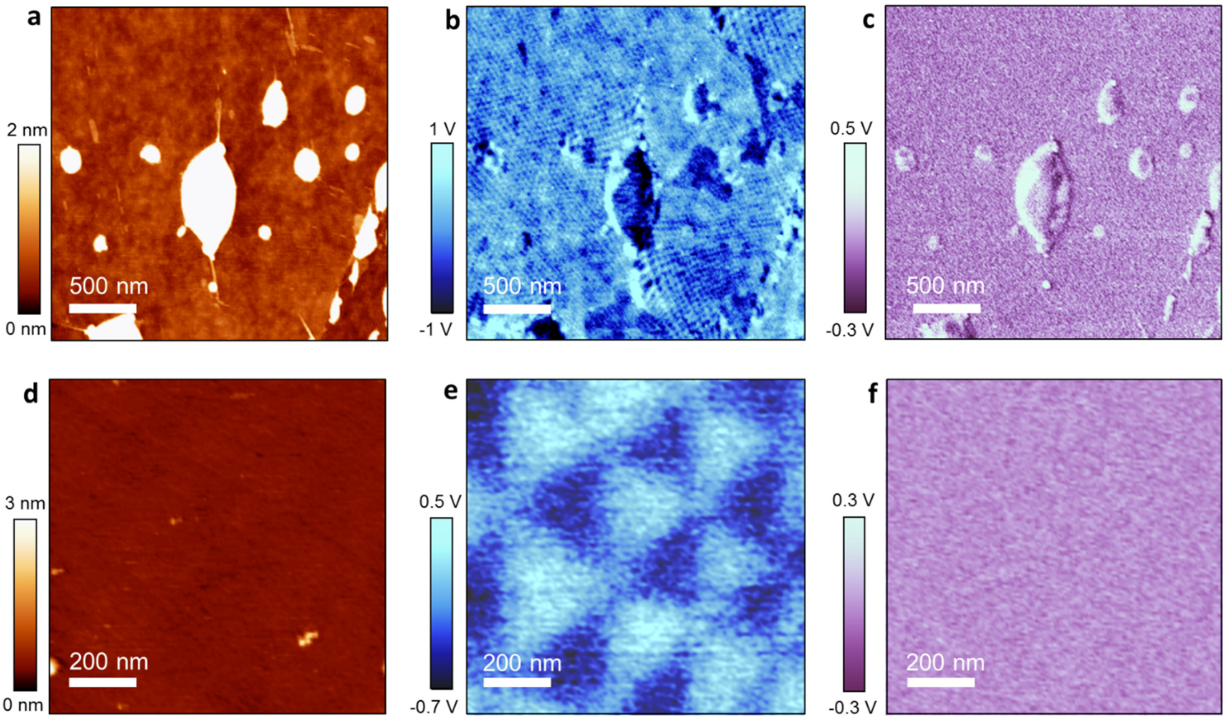

Supplementary Figure 5 | ac-EFM ω and 2ω images in marginally twisted hBN. (a,d) Zoom-in AFM topography images of Supplementary Fig. 4 and **(b,e)** corresponding ac-EFM images at ω (surface potential image) and **(c,f)** ac-EFM images at 2ω (dielectric image). A triangular potential modulation is visible in the flat regions only in in the ac-EFM images at ω in **(b,e)**. No contrast was detected in the dielectric images in **(c,f)**.

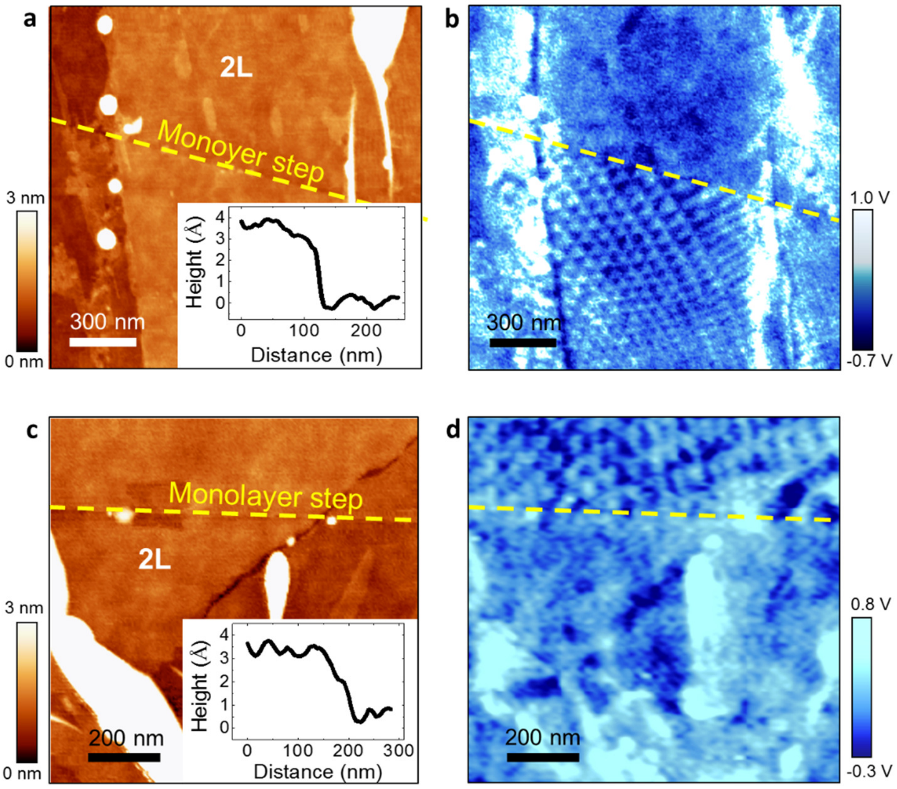

Supplementary Figure 6 | Additional EFM images of potential modulation at monolayer terraces. (a,c) AFM topography images in twisted-hBN in regions around monolayer steps, marked by the yellow dashed lines, and **(b,d)** corresponding ac-EFM images at ω. Insets: the step profiles. A triangular potential modulation is observed only on one side of the steps.

Supplementary Note 3. Experimental quantification of potential modulation and KPFM imaging

We quantified the triangular potential modulation, ΔV_s, in Fig. 3f by measuring the AFM phase shift, $\Delta\varphi$, as a function of the applied dc bias in the centre of two neighbouring domains, as shown in Supplementary Fig. 7. The observed $\Delta\varphi(V)$ curves (Supplementary Fig. 7c), which were acquired at the same scan height, are parabolic with same curvature set by the capacitive term $\partial^2 C/\partial z^2$. On the other hand, the maximum of the parabola shifted horizontally with the domain polarity, indicating a change in surface potential[6-8]. We thus quantified ΔV_s as the difference between the maximum of the two parabolas. The horizontal shift of the parabola also explains the contrast inversion upon changing the sign of the dc bias (Supplementary Fig. 7a and b). We found ΔV_s = 240 ± 30 mV on all our samples. The value was robust against variations in the domain shape, orientation and size, from micrometre range down to ~ 30 nm, the smallest domain we could detect within our resolution. For largest domains (1 μm range) with irregular shape, the domain size in Fig. 3f indicates the smallest side of the domain. The extracted ΔV_s was also independent of the number of layers in the hBN crystals (within the range of thickness studied here). Importantly, we confirmed the observed value of ΔV_s across different samples and using different AFM probes, thus proving the generality of our observation. We note that the maximum of the parabolas can shift with changing the sample, which in turn makes the quantification of the surface potential, V_s, difficult. However, what we quantified here and compared between samples is the variation of the potential, ΔV_s, not its absolute value, by measuring the shift between pairs of parabolas taken on the same sample. Such variation is robust against experimental conditions of different samples. In particular, it is independent of the cleanness of the surface, which directly affects V_s. We found appreciable changes in ΔV_s only when we brought the tip into contact or near contact with the surface, causing charge injection. Thus, the data in Fig. 3f were taken by carefully avoiding tip-surface contact.

It is important to note that the EFM contrast is not expected to depend on the moiré size using the experimental conditions used here, and this is confirmed by the experimental data in Fig. 3f. This is justified by the fact that

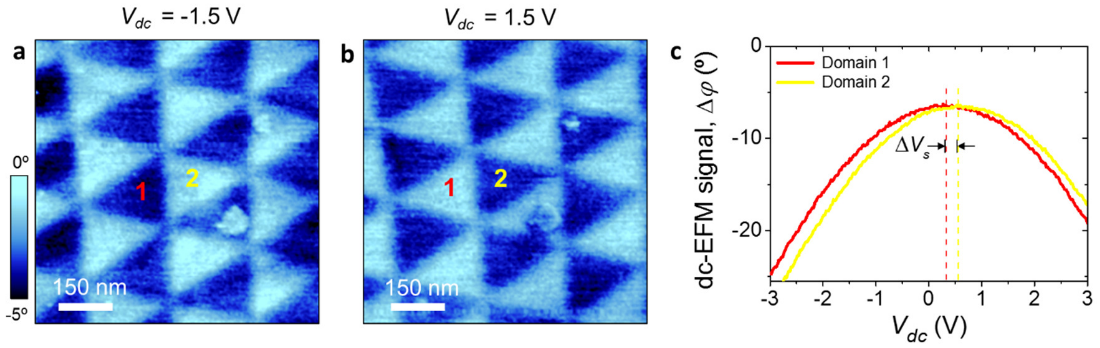

Supplementary Figure 7 | Experimental potential variation using dc-EFM. (a,b) Representative dc-EFM images on marginally twisted hBN with -1,5 V and +1.5 V dc bias applied between the tip and the sample substrate. The triangular contrast reverses upon changing the sign of the dc bias. **(c)** dc-EFM signal, $\Delta\varphi$, as a function of the applied dc bias in the centre of two neighbouring domains in **(a)** and **(b)**, marked as 1 and 2. The two curves show a parabolic behaviour with same curvature, but they are shifted in the horizontal direction, which indicates a variation in the surface potential. The dashed lines are a guide to the eye.

we took the EFM images by scanning a sharp AFM tip (radius R ≈ 5 nm, cone angle θ≈ 10º) very near to the domains plane (scan height h_s ≈ 10 nm). In these experimental conditions, the EFM contrast originates in the short-range interaction of the tip with the surface area just below and around the apex. At the same time, the domains that we analysed here were much larger than or approximately equal to 30 nm in lateral size, that is, much larger than the characteristic size of the tip. Therefore, the field lines were not sensitive to the lateral extension and shape of domains larger than few tens of nm (see Ref.[10]). It is important to note that the EFM contrast can be size-dependent on the smallest domains (30 nm < L < 100 nm) depending on the scan height, h_s, set during the EFM image. Therefore, the value of h_s needs to be chosen carefully. This is because on the smallest domains the built-in potential decays rapidly for values of h_s typically used in EFM/KPFM (tens of nm). We have experimentally determined how the EFM contrast decays when the distance between the tip and the sample become comparable with the size of the domains. Supplementary Figure 8 shows the experimental potential difference, ΔV_s as a function of h_s for different domain sizes. For large domains (L ≈ 180 nm and 600 nm), ΔV_s remained constant up to tens of nm heights, irrespectively of the domain size and shape, as expected. For the smallest domain size (L ≈ 40 nm), ΔV_s matched the value measured on larger domains at small heights (h_s < 15 nm), rapidly decreased with increasing h_s and vanished for h_s equal to the domain size. Based on these data, we

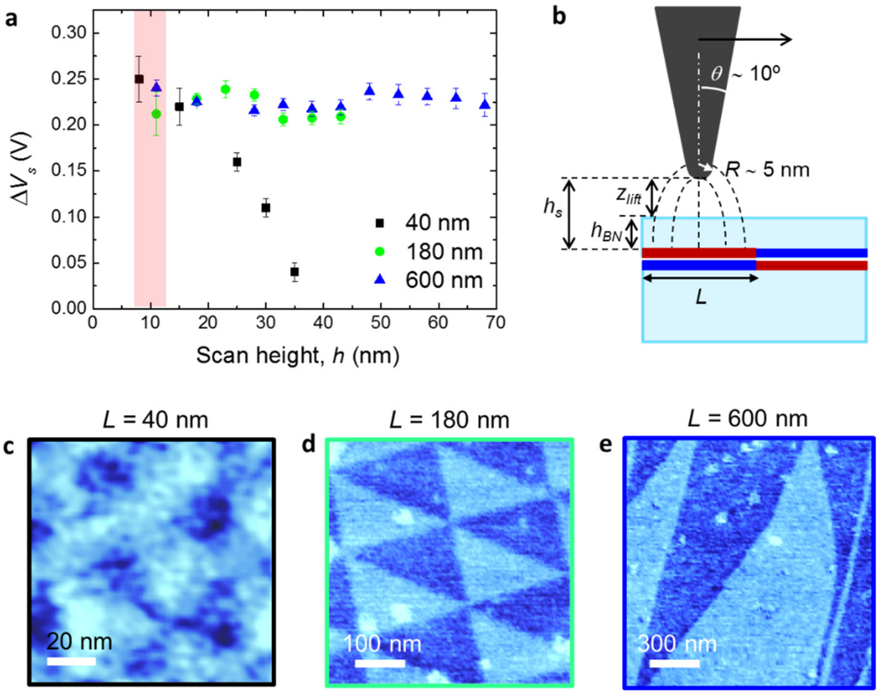

Supplementary Figure 8 | Experimental potential variation for different domain sizes. (a) Experimental electrostatic potential difference ΔV_s as a function of the scan height, h_s, for the domains shown in **(c-e)** of size L ≈ 40 nm (black squares), 180 nm (green circles) and 600 nm (blue triangles), respectively. For triangular domains of irregular shape, L indicates the smallest side of the domain. The light-red coloured region indicates the scan height at which all the data in Fig. 3f were measured. **(b)** Illustration of the AFM tip scanning over the sample with main experimental parameters. The scan height in **(a)** is the distance of the tip from the dipolar plane. **(c-e)** EFM images of the domains with increasing size measured in **(a).**

carefully measured all the domains at approximately h_s ≈ 10 nm height (light-red coloured region in Supplementary Fig. 8a), where ΔV_s is independent on the lateral size of the domains. This has guaranteed that we could detect the same potential difference for different moiré sizes down to ~ 30 nm.

To corroborate the observed value of ΔV_s, we also quantified its value by KPFM imaging[4]. To do that, we used the ac-EFM setup described above (Supplementary Fig. 3b) with an additional feedback loop and a dc bias between the tip and the silicon substrate. While the tip is scanning, the dc bias is continuously adjusted by the feedback to nullify the amplitude of the ω harmonic, now equal to $F_{el}(\omega) = \partial C/\partial z\,(V_{dc} - V_s) \cdot V_{ac}$. The KPFM image thus yields the surface potential V_s of the sample, mapping its variation across all the domains, not only in their centre. We note that also in KPFM we recorded the phase shift $\Delta\varphi(\omega)$ instead of the force to increase our lateral resolution. Supplementary Figure S9 shows representative KPFM images of the twisted-hBN sample shown in Fig. 1c, taken at the same scan height as in Fig. 3f (~ 9 nm). We found the same periodic pattern in KFPM images as in the EFM images, with large areas of regular (Supplementary Fig. 9b) and irregular (Supplementary Fig. 9a) triangular domains. The KPFM profiles (Supplementary Fig. 9c and d) show potential variations of 220-270 mV, in agreement with the value extracted from dc-EFM curves.

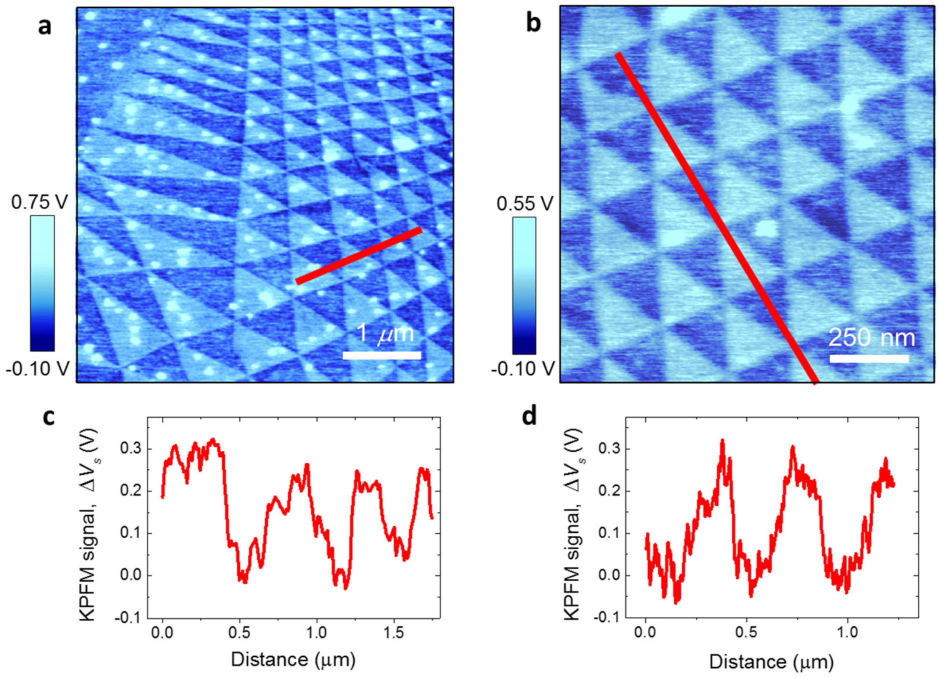

Supplementary Figure 9 | Experimental potential variation using KPFM. (a,b) KPFM images taken on the twisted-hBN sample in Fig. 1d-f in the main text. The same triangular potential pattern is detected as with dc-EFM. **(c,d)** KPFM profiles taken along the red lines in **(a)** and **(b)**, respectively. The surface potential variation between the centres of two neighbouring domains agrees with the value obtained in Fig. 3f from $\Delta\varphi(V)$ curves. Acquisition parameters: oscillation amplitude 6 nm; lift height z_{lift} = 3 nm; ac voltage bias of 4 V at 7 kHz.

Supplementary Note 4. Theoretical calculations

Theoretical results, with an additional analysis of the band structure, will be presented in Ref.[11] Here we shall just present the relevant results from that work.

Relaxation. The theoretical calculations were performed in two stages: first, we use LAMMPS[12] to minimize the energy using a classical potential model for relaxation[13], using the 'inter-layer potential'(ILP) from Refs.[14,15] with the Tersoff in-layer potential[16,17]. We minimise the positions for a supercell commensurate with the hBN one, keeping the size of the supercell fixed. Alignments are plotted using an extension of the method in Ref. [13] where we take into account all six alignment options (see Fig. 2 in the main text). This leads to a strain in all these systems that is concentrated along the zone boundaries, and gives rise to a piezoelectric charge, as shown in Supplementary Fig. 10.

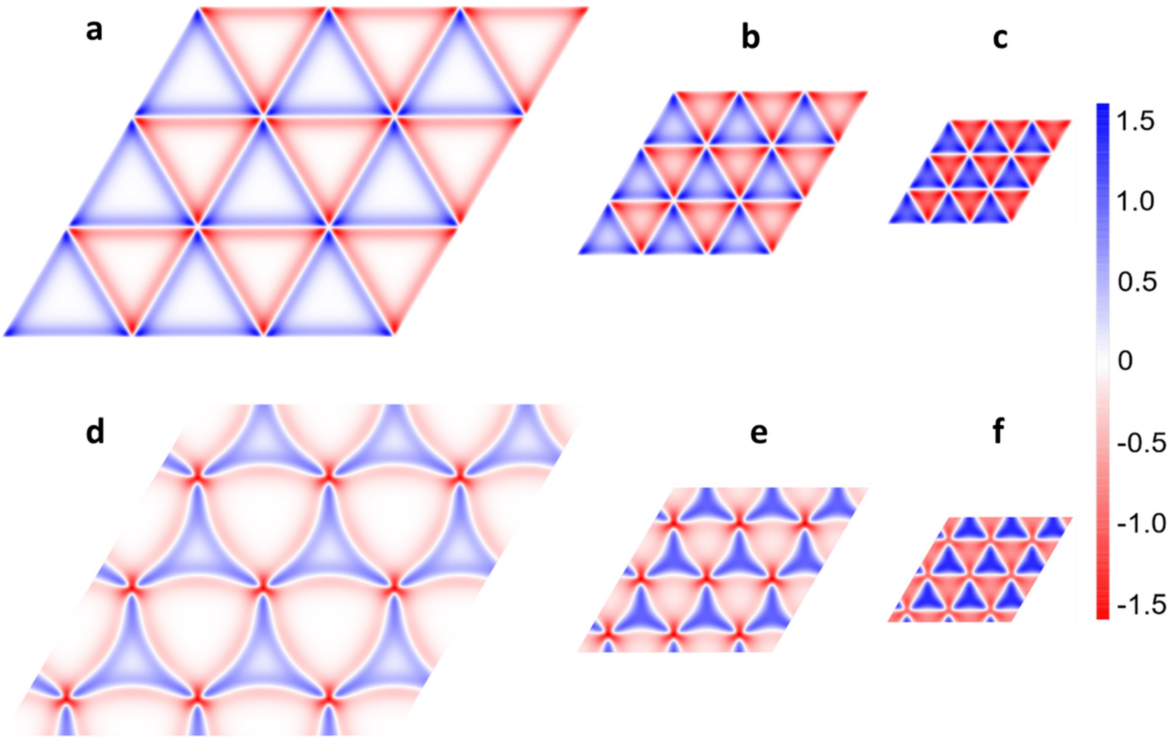

Supplementary Figure 10 | Piezo-electric charge in a relaxed hBN bilayer. The induced piezo-electric charge in a single layer after relaxation of a hBN bilayer: **(a-c)** aligned at angles **(a)** 0.33°, **(b)** 0.67° and **(c)** 1.05°; **(d-f)** anti-aligned at angles **(d)** 0.33°, **(e)** 0.67° and **(f)** 1.05°. The electron density n (scale on the right) is given in units of $10^{12}\ \mathrm{cm}^{-2}$. All images are drawn to the same scale.

Tight-binding model. Using the deformed positions, we then perform a tight-binding model. We neglect the modification of the in-layer hoppings due to the small bond stretching, and use a constant in-layer nearest neighbour hopping $t_0 = 2.33$ eV [18]. We use a simple electronic coupling using an exponential Koster-Slater interlayer model,

$$t_{XY}(r) = t_{XY} \exp(-\alpha\ (r - d),$$

where X and Y label the atomic species, $d = 0.333$ nm is the interlayer distance, and the inverse range $\alpha = 44\ \mathrm{nm}^{-1}$.

We then diagonalize the resulting tight-binding model, either for energies near the gap (which allows us to use sparse matrix techniques, and thus study much larger moirés) or by finding all states, which is required to describe the charge density. This is calculated by summing over all occupied states, which limits the smallest angle we can perform calculations for to about 1°. Further details of these calculations, as well as further results on the electronic structure of hBN can be found in Ref.[11] .

We note that in contrast to the experimental results, all our theoretical results are for a bilayer system. However, we have shown[19] that relaxation in multilayer systems still shows a sizeable reconstruction at the interface. Nevertheless, we expect that the results we get for the piezoelectric charge may be a substantial overestimate of their real magnitude.

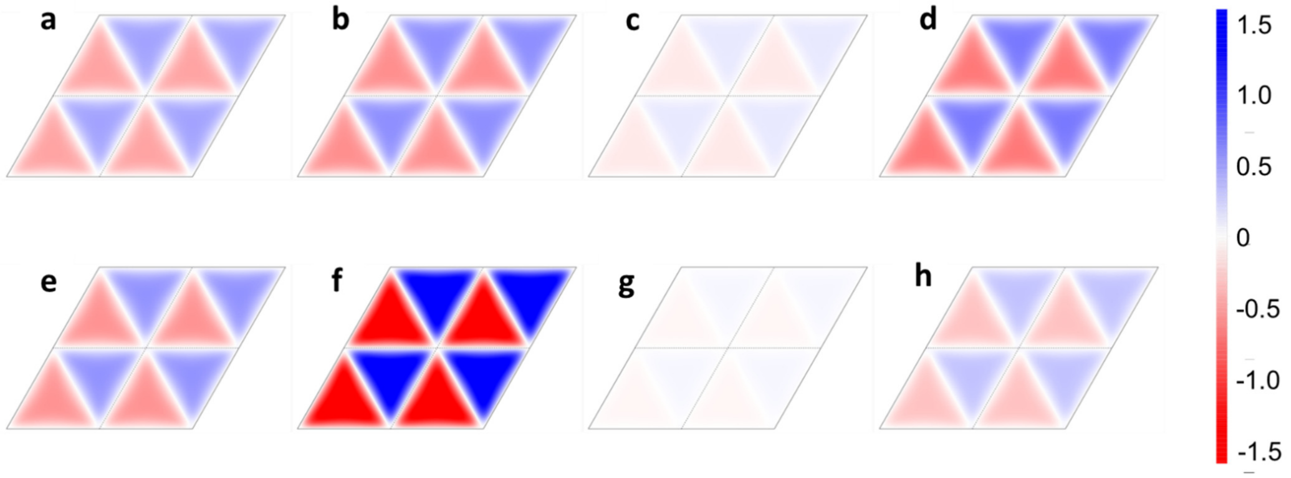

Supplementary Figure 11 | Charge density in the top-layer of twisted hBN for parallel alignment. Twist induced charge density at neutrality in the top layer for θ= 1.05° for a relaxed layer for change to the basic parameter choice $t_{BB} = 0.7$, $t_{NB} = 0.3$, $t_{NN} = 0.15$, $\Delta = 8\,\mathrm{eV}$, $\alpha = 44\,\mathrm{nm}^{-1}$ **(a)** basic parameters; **(b)** $t_{BB} = 0.5\,\mathrm{eV}$; **(c)** $\alpha = 22\,\mathrm{nm}^{-1}$; **(d)** $\alpha = 66\,\mathrm{nm}^{-1}$; **(e)** $\Delta = 6\,\mathrm{eV}$; **(f)** $t_{NB} = 0.5$ eV; **(g)** $t_{NB} = 0.15$ eV; **(h)** $t_{BB} = 1.0$ eV. The units are the same as in Supplementary Fig. 10 ($10^{12}\ \mathrm{cm}^{-2}$).

Supplementary References

1 Castellanos-Gomez, A. *et al.* Deterministic transfer of two-dimensional materials by all-dry viscoelastic stamping. *2D Mater.* **1**, 011002 (2014).

2 Frisenda, R. *et al.* Recent progress in the assembly of nanodevices and van der Waals heterostructures by deterministic placement of 2D materials. *Chem. Soc. Rev.* **47**, 53-68 (2018).

3 Kalinin, S. V. & Gruverman, A. *Scanning probe microscopy: electrical and electromechanical phenomena at the nanoscale*. Vol. I (Springer Science & Business Media, 2007).

4 Sadewasser, S. & Glatzel, T. *Kelvin probe force microscopy, measuring and compensating electrostatic forces*. (Springer, Berlin, Heidelberg, 2012).

5 Lei, C. H., Das, A., Elliott, M. & Macdonald, J. E. Quantitative electrostatic force microscopy-phase measurements. *Nanotechnology* **15**, 627-634 (2004).

6 Datta, S. S., Strachan, D. R., Mele, E. J. & Johnson, A. T. C. Surface potentials and layer charge distributions in few-layer graphene films. *Nano Lett.* **9**, 7-11 (2009).

7 Burnett, T., Yakimova, R. & Kazakova, O. Mapping of local electrical properties in epitaxial graphene using electrostatic force microscopy. *Nano Lett.* **11**, 2324-2328 (2011).

8 Minj, A. *et al.* Assessment of polarity in GaN self-assembled nanowires by electrical force microscopy. *Nano Lett.* **15**, 6770-6776 (2015).

9 Fumagalli, L., Esteban-Ferrer, D., Cuervo, A., Carrascosa, J. L. & Gomila, G. Label-free identification of single dielectric nanoparticles and viruses with ultraweak polarization forces. *Nature Mater.* **11**, 808-816 (2012).

10 Gomila, G., Gramse, G. & Fumagalli, L. Finite-size effects and analytical modeling of electrostatic force microscopy applied to dielectric films. *Nanotechnology* **25**, 255702 (2014).

11 Walet, N. R. & Guinea, F. Flat bands, strains, and charge distribution in twisted-bilayer hBN. Preprint at http://arxiv.org/abs/2011.14237 (2020)

12 Plimpton, S. Fast parallel algorithms for short-range molecular dynamics. *J. Comput. Phys.* **117**, 1-19 (1995).

13 Guinea, F. & Walet, N. R. Continuum models for twisted bilayer graphene: effect of lattice deformation and hopping parameters. *Phys. Rev. B* **99**, 205134 (2019).

14 Leven, I., Maaravi, T., Azuri, I., Kronik, L. & Hod, O. Interlayer potential for graphene/h-BN heterostructures. *J. Chem. Theory Comp.* **12**, 2896-2905 (2016).

15 Maaravi, T., Leven, I., Azuri, I., Kronik, L. & Hod, O. Interlayer potential for homogeneous graphene and hexagonal boron nitride systems: reparametrization for many-body dispersion effects. *J. Phys. Chem. C* **121**, 22826-22835 (2017).

16 Tersoff, J. New empirical approach for the structure and energy of covalent systems. *Phys. Rev. B* **37**, 6991-7000 (1988).

17 Tersoff, J. Modeling solid-state chemistry: Interatomic potentials for multicomponent systems. *Phys. Rev. B* **39**, 5566-5568 (1989).

18 Ribeiro, R. M. & Peres, N. M. R. Stability of boron nitride bilayers: Ground-state energies, interlayer distances, and tight-binding description. *Phys. Rev. B* **83**, 235312 (2011).

19 Cea, T., Walet, N. R. & Guinea, F. Twists and the electronic structure of graphitic materials. *Nano Lett.* **19**, 8683-8689 (2019).
